# Bingöl Pollen Self-Assembled Natural Thin Films: Fabrication, Characterization, and Corrosion Inhibition Performance for Copper Protection in a NaCl Environment

**DOI:** 10.1021/acsomega.5c01646

**Published:** 2025-05-27

**Authors:** Ramazan Solmaz, Ece Altunbaş Şahin, Yeşim Aydın Dursun, Yakubu Sawadogo Adam, İbrahim Halil Gecibesler, Mustafa Doğrubaş, Nevzat Çağlayan, İbrahim Y. Erdoğan, Sinan Bayindir, Gülfeza Kardaş

**Affiliations:** † 162312 Bingöl University, Health Sciences Faculty, Occupational Health and Safety Department, 12000 Bingöl, Türkiye; ‡ Bingöl University, Genç Vocational School, Property Protection and Security Department, Civil Defense and Firefighting Program, 12000 Bingöl, Türkiye; § Bingöl University, Graduate School of Natural and Applied Sciences, Chemistry Department, 12000 Bingöl, Türkiye; ∥ 37506 Çukurova University, Science and Letters Faculty, Chemistry Department, 01330 Adana, Türkiye; ⊥ Bingöl University, Graduate School of Natural and Applied Sciences, Occupational Health and Safety Department, 12000 Bingöl, Türkiye; # Bingöl University, Vocational School of Food, Agriculture, and Livestock, Plant and Animal Production Department, Beekeeping Program, 12000 Bingöl, Türkiye; ∇ Bingöl University, Science and Letters Faculty, Chemistry Department, 12000 Bingöl, Türkiye

## Abstract

This study investigates the fabrication of Bingöl pollen (B-pollen) self-assembled films (B-pollen/SAM) on Cu and their corrosion protection performance in a 3.5% NaCl solution. The films were characterized using scanning electron microscopy (SEM), atomic force microscopy (AFM), and contact angle studies. Various electrochemical techniques were employed to examine the influence of some film preparation parameters, including solvent, film formation time, and B-pollen concentration, on film quality and corrosion inhibition efficiency. The characterization results showed that orange-brown films formed on the Cu surface, exhibiting strong adhesion to the metal, and had a highly homogeneous distribution. The films effectively reduced the corrosion rate of copper in a 3.5% NaCl solution. The quality and corrosion inhibition efficiency of the films depend on the preparation conditions. The optimal B-pollen/SAM film for inhibiting copper corrosion was prepared using a 1000 ppm B-pollen solution in water with an assembly time of 24 h. Potentiodynamic polarization, electrochemical impedance spectroscopy (EIS), and linear polarization resistance (LPR) data indicated that the protection efficiencies reached 98.45, 97.2, and 97.7%, respectively.

## Graphical abstract

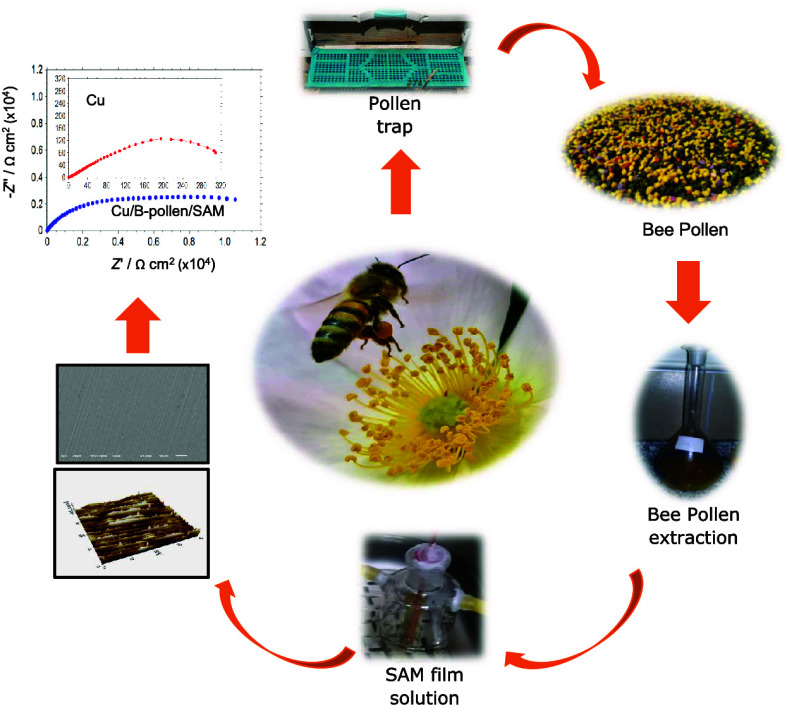

## 1 Introduction

Copper is widely used in various electronic and industrial applications, including construction materials, transportation, chemical processing, and machinery.^1–4^ Copper has excellent electrical and thermal conductivity, ease of mechanical processing, cost-effectiveness, and relative chemical inertness.^5–8^ Due to these properties, copper is extensively utilized in the microelectronics industry, especially in flexible circuits and connectors, as well as for efficient heat dissipation in high-performance processors.

Corrosion poses a significant threat to the integrity of metallic materials in various industries, prompting ongoing efforts to develop effective protection strategies.^9,10^ Although copper is a noble metal, it remains susceptible to corrosion, particularly in atmospheric conditions, in the presence of gases such as SO_2_, NO_2_, Cl_2_, and in acidic, basic, or chloride-rich environments.^11,12^ The occurrence of corrosion limits the industrial applicability of copper. Several methods have been employed to protect metals against corrosion. Cathodic protection or anodic protection,^13–15^ metal coatings, polymer films, and organic or inorganic corrosion inhibitors^16–19^ are commonly favored to protect metals against corrosion and thereby extend their service life. Organic corrosion inhibitors are often effective due to their ability to adsorb onto metal surfaces through heteroatoms and π-electron systems.^20^ However, their environmental impact has driven interest toward sustainable green alternatives.^21–24^

Bee pollen is collected from beehives and consists of a complex mixture of flower nectar, pollen, enzymes, and salivary secretions from honeybees.^25^ The composition of pollen is strongly influenced by climate conditions, geographical location, and botanical sources utilized by bees during collection.^26^ Bee pollen contains a diverse array of secondary metabolites including carbohydrates, proteins, fatty acids, polyphenols, carotenoid pigments, vitamins, phytosterols, enzymes, and coenzymes.^27^ These bioactive compounds endow pollen with antioxidant, antibacterial, antiatherosclerotic antifungal, anticancer, antiallergic, hepatoprotective chemopreventive, and immunomodulatory properties.^28^ Due to these beneficial effects, bee pollen is widely utilized in healthcare,^29^ food industries,^30^ and cosmetic formulations.^31^ In addition to its significant health effects, we have recently reported the risks that may be encountered during the collection and laboratory processing of pollen, as well as the occupational health and safety considerations that need attention.^32^

Self-assembled monolayer films (SAMs) are thin organic layers that self-organize on metal surfaces, significantly reducing the corrosion rates. While SAMs prepared from synthetic organic molecules have been extensively investigated,^33–36^ those derived from natural sources are increasingly emerging as promising candidates. SAM films form when a solid metal surface is immersed in a surfactant solution.^37–41^ The adsorption process follows a two-step kinetic mechanism.^42^ In the initial stage, rapid adsorption occurs, forming approximately 80–90% of the monolayer thickness within a few minutes. The second stage involves gradual adsorption over several hours, leading to a denser and enhanced film. In this stage, solvent molecules within the monolayer are systematically displaced and diffused, contributing to the formation of a more compact protective layer. SAM films can adhere to solid surfaces and block active corrosion sites thanks to this mechanism. The interactions between organic molecules and metal surface as well as between organic molecules and solvent molecules may occur during the formation of SAM films. These intermolecular interactions can significantly increase the complexity of SAM formation.^36,43,44^ Dai et al.^40^ reported that solvent parameters, including polarity, solubility, molecular size, octanol–water partition coefficients, and viscosity significantly influence the quality of the dodecanethiol-based SAMs. However, most of the organic compounds used in the preparation of SAM films are harmful to human health and the environment. Increasing environmental awareness and regulations necessitate the use of safer natural products in this field. Studies on the use of natural product extracts in the preparation of SAM films have only recently begun, and a few have been proposed by our research group. Beyond corrosion protection, we have shown that natural SAMs have also found applications in electrolysis^45^ or fuel cell electrodes.^46^

To achieve efficient corrosion inhibition, it is essential to optimize SAM formation parameters such as temperature, solvent type, adsorbent concentration, and exposure time.^47^ In a preliminary,^48^ we have found that B-pollen/SAM films prepared in pure water enhanced the corrosion resistance of copper. Additionally, rhodanine SAM (Rh-SAM) films were prepared on copper surface in various solvents.^49^ The study showed that methanol proved to be the most suitable medium for preparing Rh-SAM film and enhancing copper protection. We have reported a comprehensive study to optimize the solvent, surfactant concentration, and film formation time for fabricating propolis SAM films on Cu.^50^ It has been shown that the most suitable Bingöl propolis SAM films for protecting copper against corrosion were prepared in ethanol containing 1000 ppm of propolis after a 24 h assembly time. Pollen and propolis possess distinct chemical properties that directly influence the properties of relevant surface films. Compared to propolis, bee pollen offers greater availability, lower cost, and more favorable film-forming capabilities, making it a superior candidate for ecofriendly corrosion protection through SAM technology. As demonstrated by the findings of this study, the B-pollen/SAM films exhibited a superior corrosion inhibition performance. Bee pollen is rich in various bioactive compounds, including flavonoids, phenolic compounds, and other antioxidants that not only serve nutritional purposes but also exhibit protective qualities against oxidative stress.^51^ These bioactive materials contribute to the corrosion inhibition mechanism by forming a protective layer on metal surfaces, which is vital for the protection of copper under corrosive conditions.^51,52^ Therefore, research emphasizes the importance of developing B-pollen-based SAM films as a green and efficient strategy for copper protection.

While bee-derived products such as honey,^53,54^ pollen,^26,55^ and propolis^56–59^ have previously been explored as natural corrosion inhibitors, their use has predominantly involved direct addition to corrosive media. In contrast, the present study introduces a novel approach, utilizing bee pollen in the form of self-assembled monolayers. This method provides enhanced surface uniformity, stronger adsorption to the metal substrate, and a superior protective performance. To the best of our knowledge, this is the first report on the use of bee pollen in SAM-based film formation for corrosion inhibition, highlighting the innovative nature and originality of the work.

In this work, a simple environmentally friendly method was employed to prepare B-pollen/SAM films on copper electrodes. The effects of the solvent type, B-pollen concentration, and assembly time on film quality and corrosion inhibition efficiency were systematically investigated. The films were characterized by various surface characterization techniques. Corrosion tests were carried out in a 3.5% NaCl solution at 298 K. The variation of open circuit potential with exposure time (*E*_ocp_ – *t*), electrochemical impedance spectroscopy (EIS), linear polarization resistance (LPR), and anodic potentiodynamic polarization studies were used to assess the protective performance of the films.

## 2 Materials and Methods

### 2.1 Preparation of Electrodes

The working electrodes were fabricated from high-purity (>99.9%) cylindrical copper wire (length: 20 cm, diameter: 3 mm). Approximately 5 cm section from one end of copper wire was insulated with polyester or Teflon, leaving only the lower tip available for electrochemical measurements. The exposed surface area of copper was 0.0707 cm^2^. For characterization purposes, samples of approximately 5 mm in length were cut and embedded in polyester (for water (W), methanol (M), ethanol (E)), or Teflon (for acetonitrile (AN), acetone (A), toluene (T), ethyl acetate (EA), toluene (T), and hexane (H)), ensuring compatibility with the instruments. Before the SAM film assembly, copper surfaces were prepared as previously described in detail.^61^ A Pt sheet with dimensions of 10 mm × 10 mm × 1 mm served as the counter electrode. A commercial Ag/AgCl reference electrode (3 M KCl) was used for all of the electrochemical measurements. All recorded potentials were referred to this reference electrode.

### 2.2 Preparation of B-Pollen/SAM-Modified Electrodes

Bee pollen samples were collected from apiaries located in the northern provinces of Bingöl and Türkiye (Adaklı, Kiǧı, Yedisu, and Yayladere) using pollen traps integrated into the beehives. During the pollen extraction process, a maceration process was employed for 72 h without heating the solution mixture. In order to optimize the film extract for achieving optimal film quality, the extraction was carried out using various solvents, e.g., W, M, E, AN, A, T, EA, H. The same solvents were utilized for the film assembly. The methodology for extraction and film assembly followed the previously described procedure for propolis extraction.^50^ The optimal B-pollen/SAM film for Cu protection was developed by systematically varying the solvent type (W, M, E, AN, A, T, EA, H), B-pollen concentration (100, 250, 500, 1000, and 2000 ppm), and film assembly time (2–72 h). The films were self-assembled in a specifically designed glass cell, which was close to the atmosphere. Approximately 30 mL of the film-forming solution, prepared with different solvents and B-pollen concentrations, was added to the cells. To remove dissolved O_2_, pure N_2_ gas was purged through the system. Polished and cleaned Cu electrodes were then immersed in the solutions for designated self-assembly periods. During immersion, N_2_ purging continued for an additional 2 h.^50^ Throughout the SAM film assembly process, the temperature was thermostatically maintained at room temperature. Once the self-assembly was complete, the electrodes were removed from the solution, carefully rinsed with the same solvent, dried under a stream of N_2_, and stored in a desiccator until further use in experiments.

### 2.3 Electrochemical Measurements

The corrosion behavior of uncoated Cu and SAM-modified Cu electrodes was evaluated in a 3.5% NaCl solution at 298 K using a computer-controlled CHI electrochemical workstation. Uncoated Cu and B-pollen/SAM electrodes were used as working electrodes, while Pt and Ag/AgCl (3 M KCl) served as the counter and reference electrodes, respectively. Initially, the uncoated or the B-pollen-modified Cu electrodes were immersed in the test solution for 1 h, and the *E*_ocp_ of the electrodes was recorded as a function of the immersion time. Once a steady-state open circuit was reached, EIS, LPR, and anodic potentiodynamic polarization measurements were performed. EIS studies were performed at *E*_ocp_ over a frequency range from 100 kHz to 0.03 Hz by applying a 5 mV sinusoidal wave signal. The Zwiev software was used to fit the EIS data and calculate relevant parameters. LPR studies were carried out immediately after the EIS measurements by scanning the potential ±0.01 V from more cathodic potentials around *E*_ocp_ at a scan rate of 1 mV s^–1^. Following the LPR measurements, anodic potentiodynamic polarization measurements were performed starting from *E*_ocp_ to 1.2 V at a scan rate of 1 mV s^–1^. To ensure consistency, the temperature of the solutions was maintained at 298 K using a thermostat.

### 2.4 Characterizations of B-Pollen Films

The characterizations of uncoated and B-pollen-modified Cu electrodes were performed using scanning electron microscopy (SEM) (JEOL 6510), atomic force microscopy (AFM) (Nanotechnology Park System XE-100), and contact angle (Biolin Scientific, Theta Lite) measurements. Contact angle measurements were performed using the sessile drop method at room temperature with a water drop volume of approximately 2 μL. To ensure accuracy and reliability, contact angle values were measured in three different regions on each electrode surface. These values were then averaged to obtain reliable and representative measurements.

### 2.5 Exactive Plus Orbitrap HPLC-HRMS Analysis

High-performance liquid chromatography-high-resolution mass spectrometry (HPLC-HRMS) analyses were performed using a Thermo Fisher Scientific Exactive Plus Orbitrap HRMS system, equipped with a heated electrospray ionization (HESI) interface, a column furnace, an autosampler, and a Dionex UltiMate 3000 RS pump. The Orbitrap-MS instrument was calibrated by using both positive and negative ionization mode calibration solutions via an automated syringe injector. The TraceFinder 3.2 software was utilized to control and operate both the mass spectrometry (MS) and liquid chromatography (LC) systems simultaneously, while Xcalibur software was used for data acquisition and processing. Separation was carried out using a 3 μm NX-C18 Phenomenex Gemini column, maintained at a constant temperature of 30 °C. The mobile phase consisted of pure methanol (mobile phase B) and glacial acetic acid in water (mobile phase A), applied in a programmed elution gradient.^60^

## 3 Results and Discussion

### 3.1 Corrosion Tests

#### 3.1.1 Variation of Open Circuit Potential with Exposure Time

A literature survey has shown that the properties of SAM films are notably influenced by the type of solvent or solvent polarity used.^61,62^ Therefore, the variation in the open circuit potentials (*E*_ocp_) of uncoated and Cu/B-pollen/SAM-modified electrodes, assembled in different solvents (W, E, M, A, AN, EA, T, and H), was recorded as a function of exposure time, and the data obtained are presented in Figure 1a. During solvent optimization, the B-pollen concentration was kept at 1000 ppm, and the assembly time was set to 24 h. It is important to note that achieving a stable *E*_ocp_ is critical for obtaining accurate and reliable data. The stability of the *E*_ocp_ value implies that the adsorption–desorption equilibrium at the metal/electrolyte interface is approaching completion. Furthermore, this variation provides valuable insights into the initiation and progression of the corrosion process, as well as the dominant of anodic or cathodic mechanisms involved.^63,64^

**Figure 1:**
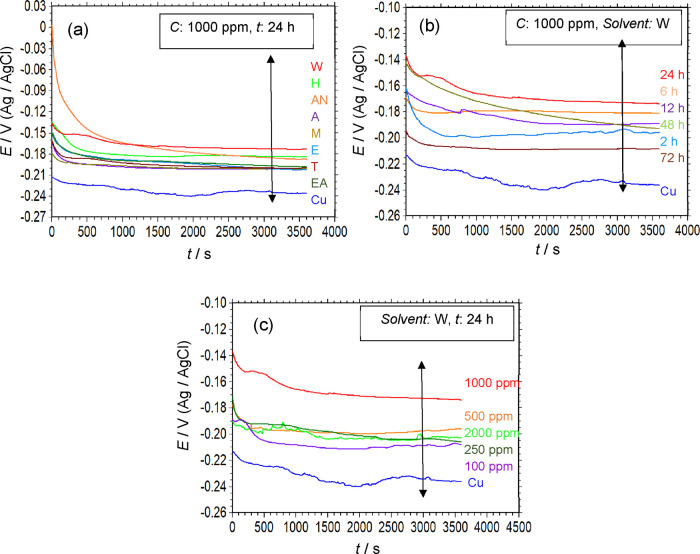
Change of *E*_ocp_ in a 3.5% NaCl solution as a function of exposure time for the uncoated Cu and B-pollen/SAM-modified Cu electrodes, which were modified under various conditions; different solvents (a), film assembly times (b), and B-pollen concentrations (c).

Figure 1a shows that the *E*_ocp_ of the uncoated Cu electrode ranges from −0.211 to −0.236 V, remaining nearly stable after 1 h of exposure. As shown in Figure 1a, the SAM-modified films assembled in different solvents exhibit more anodic *E*_ocp_ values with respect to the unmodified Cu electrode. This observation suggests that the SAM films act as a physical barrier, primarily influencing the anodic reaction. The differences in *E*_ocp_ values of the film-modified electrodes can be related to variations in the quality of the surface film, which are likely influenced by the choice of solvent. However, no clear correlation was observed between the solvent polarity and *E*_ocp_ variation.

The composition of the pollen extract significantly depends on the type of solvent used, which, in turn, affects the chemical composition and the quality of the resulting film. Among the tested solvents, the most anodic *E*_ocp_ was observed for the film assembled in water (W) after a 1 h exposure, compared to the other SAM-modified electrodes. The assembly process of these films begins with the orientation of the molecules onto the metal surface, followed by their adsorption. In the final step, they undergo reorganization to form a high-quality surface film. Therefore, the observed difference in *E*_ocp_ values across different solvents can be explained by variations in the molecular assembly mechanisms, orientation, and adsorption rates of the B-pollen molecules on the copper surface.

Further electrochemical studies (see Sections 3.1.2–3.1.4) revealed that using W as a solvent for film assembly resulted in the maximum corrosion resistance. Consequently, the film assembly time and concentration optimizations for preparing Cu/B-pollen/SAM-modified electrodes were carried out in this solvent.

Figure 1b shows the *E*_ocp_ – *t* curves of B-pollen-modified electrodes prepared at various film assembly times in water, using a 1000 ppm of B-pollen solution. The *E*_ocp_ values of the SAM-modified electrodes were more positive than those of uncoated copper, further supporting the idea that the SAM films predominantly influence the anodic reaction. The film acts as a simple barrier between the corrosive medium and the copper surface, limiting the diffusion of aggressive species to the copper surface.

In general, as the assembly time increased, *E*_ocp_ shifted to more positive values. However, after 24 h of film formation time, the *E*_ocp_ shifted slightly back to cathodic potentials. This change is probably related to variations in the properties of the film formed on the surface. Further experimental studies are required to fully elucidate this phenomenon. Notably, the more positive *E*_ocp_ was observed for the film assembled over a 24 h period.

The *E*_ocp_ – *t* curves of B-pollen/SAM-modified electrodes prepared using different B-pollen concentrations (100–2000 ppm) in water after a 24 h assembly time are shown in Figure 1c. Similar to the trends observed in Figure 1a,b, the *E*_ocp_ values of the film-modified electrodes remain nobler than those of the uncoated copper electrode. The *E*_ocp_ value of the SAM films prepared in 1000 ppm of B-pollen solution is notably more positive than those prepared at other concentrations. This observation suggests that at 1000 ppm, B-pollen molecules effectively attach to the copper surface, leading to the formation of a high-quality protective film. Conversely, at higher concentrations (e.g., 2000 ppm), the *E*_ocp_ moved to more negative potentials but still remained nobler than that of the uncoated copper electrode. This trend can be explained by changes in the structure of the surface film, which likely reduce its quality and uniformity. Supporting evidence from SEM and AFM studies indicates that excessive B-pollen concentration may lead to the formation of a less compact and less protective surface layer.

#### 3.1.2 Potentiodynamic Polarization Studies

Key kinetic parameters related to corrosion can be derived from potentiodynamic polarization plots. The anodic potentiodynamic polarization curves of the uncoated and B-pollen/SAM-modified Cu electrodes, prepared under different assembly conditions, are shown in Figure 2. The curves were obtained in a 3.5% NaCl solution after a 1 h exposure. The related electrochemical parameters, including *E*_corr_, *i*_corr_, anodic Tafel slopes (*b*_a_), percentage corrosion inhibition efficiency (η%), current densities at 0.100 V (Ag/AgCl) (*i*_0.100 V_), and at 0.150 V (Ag/AgCl) (*i*_0.150 V_), were calculated from these curves, and results are shown in Table 1. The formula given below was used to determine the corrosion inhibition efficiency (η%)

1 $$\eta \%=\left(\frac{i_{\mathrm{cor}}^{\prime }-i_{\mathrm{cor}}}{i_{\mathrm{cor}}^{\prime }}\right)\times 100$$

Here, *i*_cor_′ and *i*_cor_ represent the corrosion current densities of uncoated and SAM-modified Cu electrodes, respectively.

**Figure 2:**
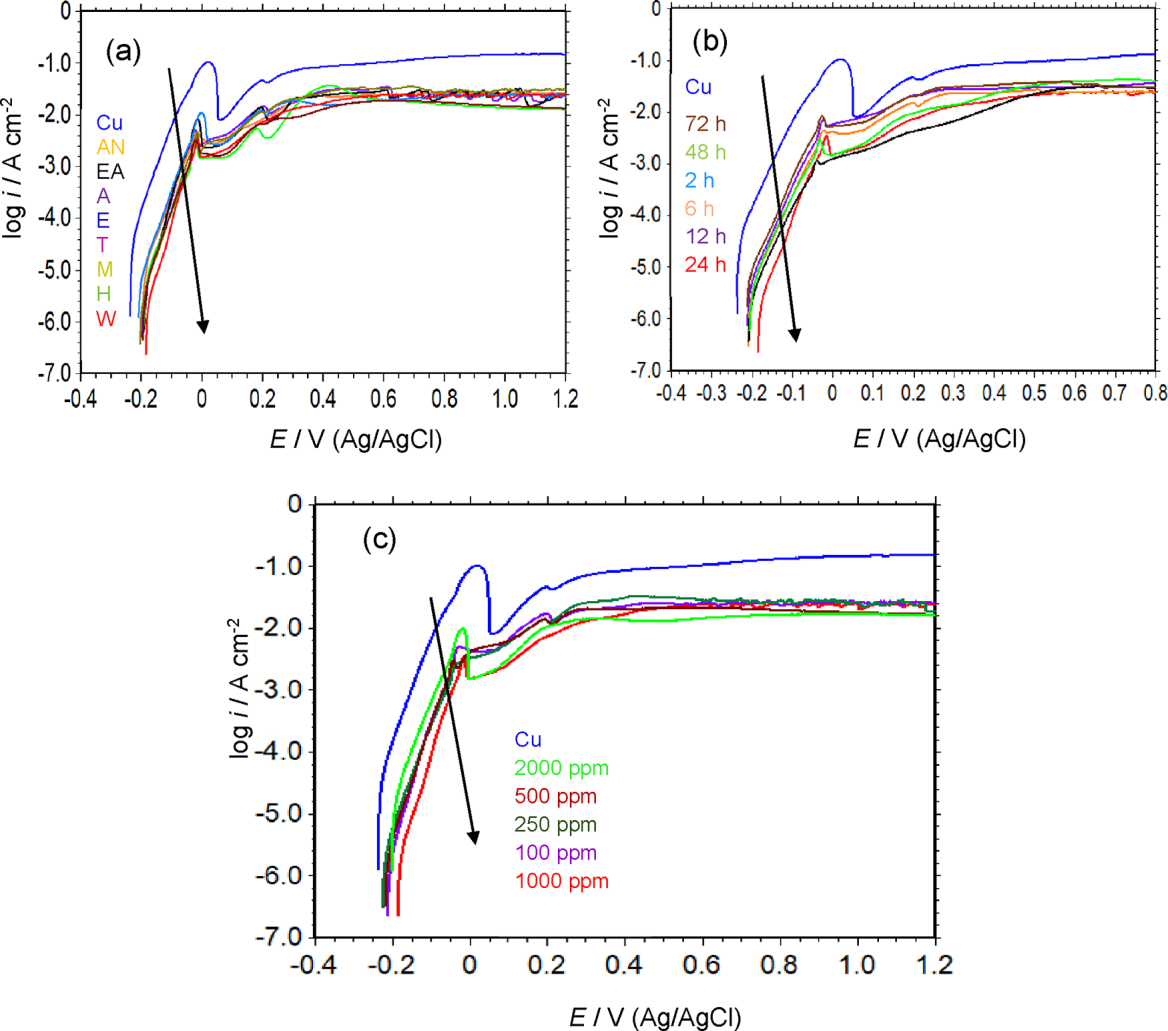
Anodic potentiodynamic polarization curves of the uncoated Cu and B-pollen/SAM-modified Cu electrodes, which were modified under various conditions; different solvents (a), film assembly times (b), and B-pollen concentrations (c). The curves were recorded in a 3.5% NaCl solution following a 1 h immersion period.

**Table 1:** Electrochemical Parameters of the Uncoated Cu and B-Pollen/SAM-Modified Cu Electrodes, Assembled under Various Pollen Concentrations, Solvents, and Film Assembly Times, Calculated from Anodic Potentiodynamic Polarization Plots

film assembly conditions		*E*_corr_ (V, Ag/AgCl)	*i*_corr_ (μA cm^–2^)	*b*_a_ (mV dec^–1^)	η%
uncoated Cu		–0.236	70.75	60	
solvent optimization *t*: 24 h, *C*: 1000 ppm	E	**–**0.209	5.607	59	92.07
	**W**	**–0.185**	**1.090**	**45**	**98.45**
	M	–0.204	2.024	55	97.14
	AN	–0.194	9.422	63	86.68
	A	–0.198	2.468	49	96.51
	EA	–0.190	3.161	47	95.53
	T	–0.194	3.869	59	94.53
	H	–0.190	6.826	69	90.35
concentration optimization *t*: 24 h, solvent: W	100 ppm	–0.212	11.151	46	98.37
	250 ppm	–0.224	2.309	60	96.73
	500 ppm	–0.217	1.613	52	97.72
	**1000 ppm**	**–0.185**	**1.090**	**45**	**98.45**
	2000 ppm	–0.201	1.159	65	97.74
assebly time optimization *C*: 1000 ppm, solvent: W	2 h	–0.211	3.658	46	94.82
	6 h	–0.209	3.286	59	95.35
	12 h	–0.208	2.471	62	96.50
	**24 h**	**–0.184**	**1.090**	**45**	**98.45**
	48 h	–0.205	4.367	62	93.82
	72 h	–0.211	4.907	55	93.06

As shown in Figure 2 and Table 1, the *E*_ocp_ value of the unmodified Cu electrode was −0.236 V, consistent with the *E*_ocp_ – *t* plots. By scanning the potential from *E*_ocp_ toward more anodic potentials, a rapid increase in current density was observed. Between −0.210 and −0.06 V, the variation of log *i* versus applied potential was nearly linear, indicating that the electrode exhibits Tafel behavior in this environment. This suggests that the Cu dissolution in the NaCl solution is activation-controlled.^65–67^

The observed current density results from the conversion of Cu to Cu­(I). A current peak was observed at 0.02 V, followed by a decrease in current density until +0.06 V. This decline can be attributed to the precipitation of CuCl, which has a low solubility on the metal surface. Beyond this potential, the current density increased again due to the formation of soluble cuprous chloride complex, CuCl_2_^–^ species. After +0.215 V, the diffusion-controlled current limit was reached due to the formation of soluble Cu­(II) species and their subsequent diffusion from the metal/solution interface. The main cathodic reactions occurring in this condition involve the reduction of dissolved oxygen.^68–70^ The proposed dissolution reaction mechanism of Cu in 3.5% NaCl solutions can be expressed by the following reactions^49,50,69,70^

2 $$\mathrm{Cu}+{\mathrm{Cl}}^{-}↔{\mathrm{CuCl}}_{\mathrm{ads}}+e^{-}\left(\mathrm{fast step}\right)$$

3 $${\mathrm{CuCl}}_{\mathrm{ads}}+{\mathrm{Cl}}^{-}↔{\left[{\mathrm{CuCl}}_{2}\right]}^{-}\left(\mathrm{rate determining}\right)$$

4 $${\left[{\mathrm{CuCl}}_{2}\right]}^{-}\to {\mathrm{Cu}}^{2+}+{2\mathrm{Cl}}^{-}+e^{-}$$

The polarization curves of SAM-modified electrodes showed almost similar behavior to those of uncoated copper. Both Figure 2a and Table 1 clearly show that the current densities of modified electrodes were significantly reduced, and the *E*_corr_ shifted toward more positive potentials when a B-pollen thin film was assembled on Cu from all solvents. This shift indicates that the SAM films primarily influence the anodic reaction mechanism.

The polarization plots in the Tafel region were nearly parallel, and the *b*_a_ values did not change significantly, suggesting that the films do not alter the metal dissolution mechanism but act as a physical barrier between the metal surface and the corrosive medium. The corrosion protection efficiency of the film varied depending on the solvent used during film assembly, ranging from 86.68 to 98.45%.

No distinct correlation was observed between solvent polarity and the corrosion protection ability. However, the chemical composition of the pollen extract as well as surface SAM film strongly depends on the type of solvent, which may contribute to variations in the inhibition performance. However, further studies are required to clarify this relationship, which could be explored in future research.

Notably, when water was used as the solvent for film assembly, *i*_corr_ decreased from 70.75 μA cm^–2^ (uncoated Cu) to 1.09 μA cm^–2^, while *E*_corr_ moved 51 mV toward more anodic potentials. The superior corrosion inhibition performance obtained in water can be attributed to the better quality of the surface film. Consequently, this solvent was selected for further film optimization studies.

Figure 2b shows the anodic potentiodynamic polarization curves of the B-pollen/SAM-modified electrode, which were assembled for different film assembly times in water at a constant pollen concentration. The electrodes modified under varying assembly times showed similar to that observed in the solvent optimization study. As evident from Figure 2b and Table 1, the current density decreased and the protective performance of the film increased as the film assembly time increased. The highest corrosion mitigation ability was achieved when the B-pollen/SAM films were assembled for a 24 h exposure. Beyond this time, a further increase in film formation time resulted in an increase in current density and a decline in protection efficiency.

A similar trend was observed for *E*_corr_, which shifted toward more anodic potentials up to 24 h of film assembly time and then moved slightly toward more cathodic values with extended assembly time. However, all SAM-modified electrodes remained more anodic than the uncoated Cu electrode. The observed reduction in the anodic current density and the shift in *E*_corr_ toward more anodic potentials confirm that all films act as anodic inhibitors. The *b*_a_ values remained nearly unchanged, indicating that the film reduces the corrosion current density without altering the metal dissolution mechanism.

As previously mentioned, SAM formation includes three key steps: adsorption, molecular rearrangement, and stabilization. Each step requires a specific duration to achieve a stable, homogeneous film structure. The optimal corrosion protection obtained after 24 h suggests that this is the necessary time for the self-assembly process to reach equilibrium. Based on these findings, 24 h was identified as the optimal film assembly time for B-pollen/SAM coatings to protect copper in a 3.5% NaCl solution, and this condition was selected for further studies.

The anodic potentiodynamic polarization curves of the Cu/B-pollen/SAM electrode, which were prepared at different B-pollen concentrations, are shown in Figure 2c. As shown in Figure 2c, the polarization behavior of Cu electrodes modified at varying pollen concentrations was consistent with the findings from the solvent and assembly time optimizations. Increasing pollen concentration led to a decrease in current density and a shift in *E*_corr_ toward more anodic values, confirming the anodic inhibition effect of the films. The *b*_a_ remained unchanged compared to uncoated Cu, indicating that the films act as a barrier between the metal surface and the corrosive environment without affecting the dissolution mechanism. The optimal pollen concentration for maximum corrosion protection was determined to be 1000 ppm, yielding a corrosion inhibition efficiency of 98.45%.

#### 3.1.3 Electrochemical Impedance Spectroscopy

The electrochemical behavior of copper electrodes modified with B-pollen/SAM films was studied using EIS under identical conditions. The EIS technique provides critical insights into the processes occurring at the metal/solution interface. Since these measurements can be conducted without disturbing the structure of the metal/solution interface, this method is widely used in corrosion inhibition studies and in analyzing the behavior of thin films.^71–74^

Nyquist and Bode-phase plots of the uncoated and B-pollen/SAM-modified Cu electrodes prepared under different assembly conditions are shown in Figure 3. These data were collected in a 3.5% NaCl solution at 298 K after a 1 h exposure. Numerical impedance parameters were derived by fitting experimental data using Zview software program. The electrical equivalent circuit diagrams (EECDs) proposed for this purpose are listed in Figure 4.

**Figure 3:**
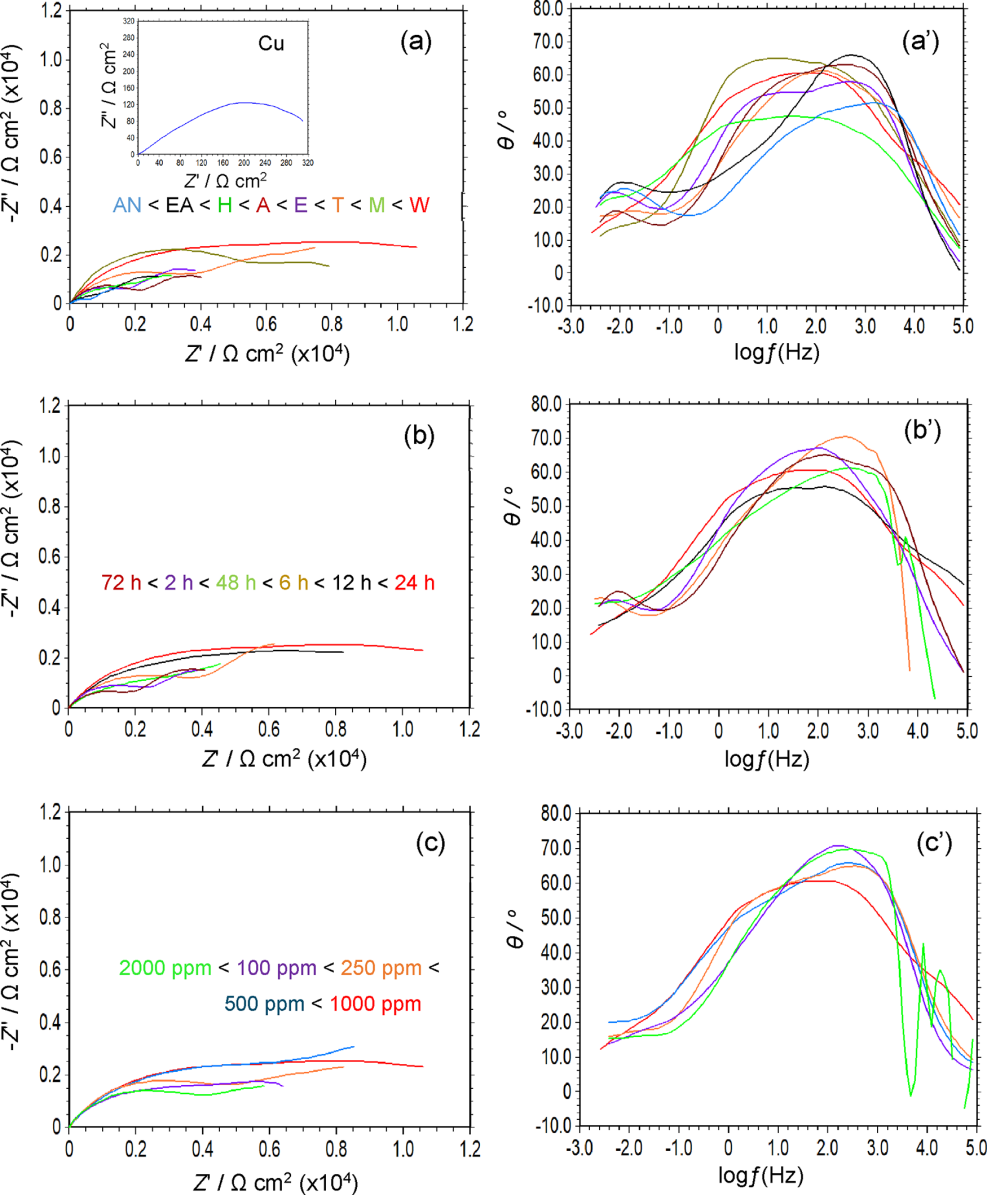
Nyquist (a–c) and Bode-phase (log *f* – θ) (a′–c′) plots of uncoated Cu and B-pollen/SAM-modified Cu electrodes assembled under varying conditions: different solvents (a, a′), film formation times (b, b′), and B-pollen concentrations (c, c′).

**Figure 4:**
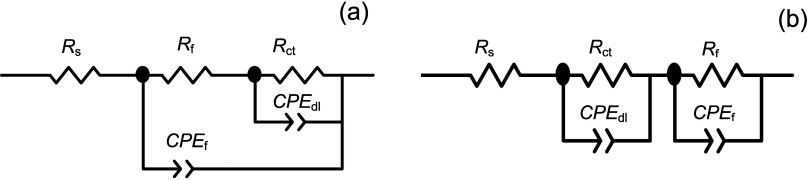
Electrical equivalent circuit diagrams proposed for the uncoated Cu/NaCl solution interface (a) and the B-pollen/SAM-modified Cu/solution interface (b).

The experimental data were fitted to these circuit models, and the associated parameters are given in Table 2. In this model, *R*_s_ is the solution resistance, *R*_ct_ is the charge-transfer resistance, *R*_d_ denotes the diffuse layer resistance, *R*_a_ accounts for the resistance of accumulated soluble corrosion products or unattached pollen molecules at the metal/solution interface, *R*_por_ represents the pore resistance associated with the resistances appearing in the pores of the film of the copper species or film, *R*_por_ (*R*_ct_ + *R*_d_ + *R*_a_) is the pore resistance, *R*_f_ corresponds to the resistance of the films formed on the metal surface, including chemically attached (assembled) layers and participated insoluble copper oxides, chlorides, or hydroxides, and *R*_p_ is given as the polarization resistance (*R*_f_ + *R*_por_), reflecting the total resistance. CPE_f_ and CPE_dl_, which contain *Y* ^o^ and *n*, also refer to a constant phase element for the film and double layer, respectively.^50,75^ The “*n*” is a measure of surface inhomogeneity and indicates deviation from an ideal capacitor (phase shift).

**Table 2:** Electrochemical Parameters of Uncoated Cu and B-Pollen/SAM-Modified Cu Electrodes, Assembled under Various Pollen Concentrations, Solvent Types, and Film Assembly Times, Calculated from the EIS Analysis

				CPE_dl_			CPE_f_				
film assembly conditions		*R*_s_ (Ω·cm^2^)	*R*_ct_ b	*Y*^o^ (10^–6^/s*^n^* Ω^–1^·cm^–2^)	*n*_dl_	*R*_f_ (Ω·cm^2^)	Y^o^ (10^–6^/s*^n^* Ω^–1^·cm^–2^)	*n*_f_	*R*_p_ (Ω·cm^2^)	η%	χ^2^
uncoated Cu		0.78	3.31	2425.2	0.73	350	12,615	0.54	353.31		0.002
solvent optimization *t*: 24 h, *C*: 1000 ppm	E	2.29	1823	210	0.69	4571	4571	0.46	6394	94.4	0.006
	**W**	**2.49**	**2841**	**169**	**0.82**	**9827**	**477**	**0.58**	**12,668**	**97.2**	**0.003**
	M	2.17	5044	116	0.81	6842	1500	0.56	11,886	97.0	0.001
	AN	2.22	565	232	0.63	3515	4666	0.55	5231	91.3	0.005
	A	2.08	2032	108	0.74	3453	394	0.73	5485	93.5	0.002
	EA	1.87	1046	382	0.61	2704	5070	0.79	3750	94.2	0.020
	T	3.82	3066	68	0.73	9073	939	0.56	12,139	97.0	0.001
	H	2.84	2350	625	0.57	1900	14,900	0.92	4250	91.6	0.001
concentration optimization *t*: 24 h, solvent: W	100 ppm	4.12	3908	104	0.73	4120	1636	0.78	8028	95.5	0.009
	250 ppm	2.71	6030	1860	0.73	4406	102	0.75	10,436	96.6	0.04
	500 ppm	2.74	7593	507	0.73	2470	119	0.76	10,063	96.5	0.010
	**1000 ppm**	**2.49**	**2841**	**169**	**0.82**	**9827**	**477**	**0.58**	**12,668**	**97.2**	**0.003**
	2000 ppm	12.90	5155	320	0.53	895	690	0.73	6050	94.1	0.377
assembly time optimization *C*: 1000 ppm, solvent: W	2 h	2.16	2209	160	0.77	5524	3300	0.69	7733	95.4	0.002
	6 h	11.34	2852	126	0.72	7637	1960	0.69	10,489	96.6	0.358
	12 h	2.83	2320	138	0.76	10,200	423	0.53	12,520	97.1	0.002
	**24 h**	**2.49**	**2841**	**169**	**0.82**	**9827**	**477**	**0.58**	**12,668**	**97.2**	**0.003**
	48 h	4.00	1125	124	0.76	3403	1242	0.83	4528	92.1	0.098
	72 h	1.66	1885	127	0.76	4165	4120	0.80	6050	94.1	0.006

The η% values were determined by using the following equation

5 $$\eta \%=\left(\frac{R_{p}^{\prime }-R_{p}}{R_{p}^{\prime }}\right)\times 100$$

In this equation, *R*_p_ represents the polarization resistance of the unmodified Cu electrode, while *R*′_p_ corresponds to the polarization resistance of the SAM-modified Cu electrode.

Figure 3a,a′ shows the Nyquist and Bode-phase (log* f* – θ) curves of uncoated Cu and B-pollen-modified Cu electrodes prepared in different solvents at a constant 1000 ppm B-pollen concentration and a 24 h film assembly time. The uncoated Cu data were included for comparison. As seen in the inset Nyquist plot in Figure 3a,a′, two-time constants appeared for the uncoated Cu electrode. The first time constant at high and medium frequencies was associated with CPE_dl_ – *R*_ct_, while the second one at low frequencies corresponds to the CPE_f_ – *R*_f_. The second loop suggests that the corrosion reaction at the uncoated electrode is diffusion-controlled, resulting from the diffusion of dissolved corrosion products and the diffusion of O_2_ gas from the solution side to the metal surface.^50,76^ The *R*_p_ of the uncoated Cu electrode was calculated as 320 Ω·cm^2^ in a 3.5% NaCl solution after a 1 h exposure.

As seen from Figure 3a,a′, the characteristics of the Nyquist and Bode-phase plots of the SAM-modified electrodes in different solvents are identical. However, the magnitudes of the time constants were significantly higher than those of the uncoated Cu electrode. The data in Table 2 confirms that all of the modified electrodes prepared in different solvents exhibit higher *R*_p_ values and have more than 90%, making them suitable for practical applications. The increased corrosion resistance is associated with the formation of a barrier film between the metal and corrosive medium, reducing the charge-transfer process between the metal and the solution.^49,50^ The protective efficiency of the B-pollen/SAM film depends on the assembly conditions, particularly, the solvent type. Differences in the film properties, which will be further discussed in subsequent characterization studies, likely contribute to this variation. Depending on the solvent type, the η% ranges between 91.6 and 97.2%. Figure 3a,a′ and Table 2 clearly show that the highest *R*_p_ and η% were observed when water was used as the solvent during the film assembly. The lack of distinct separation between the two loops in the Nyquist plot suggests that this film is more compact on the surface. A decrease in CPE_f_ and CPE_dl_ for the SAM-modified Cu electrodes, compared to those of the uncoated Cu electrodes, suggests that the presence of SAM films formed on the metal surface thickens the double layer according to the Helmholtz model. However, no distinct correlation was observed between the solvent polarity and the *n*_dl_ and *n*_f_ values.

A similar behavior was observed in the optimization studies for the film assembly time. Figure 3b,b′ presents the Nyquist and Bode-phase plots of B-pollen/SAM-modified electrodes prepared after various film assembly times. The related EIS parameters are shown in Table 2. Figure 3b,b′ and Table 2 suggest that the electrochemical behavior of the B-pollen-modified Cu electrodes varies with the film assembly time. As reported by Preife,^77^ the adsorbed layer begins to form within the first few hours of self-assembly. However, it lacks organization and density, leading to potential defects. Therefore, the *R*_p_ value is expected to be lower at shorter assembly times (e.g., 2 and 6 h). The *R*_p_ value of the modified Cu electrode increases up to a 24 h film assembly time, providing maximum protection. As the deposition time increases, a greater number of SAM molecules adhere to the Cu surface. This results in increased surface coverage, thickening of the film at the metal–solution interface, and ultimately leading to a reduction in CPE values.^78,79^ Beyond the 24 h film assembly time, the *R*_p_ value reduces again. As the self-assembly process of SAMs involves adsorption, rearrangement, and stabilization, each step takes place over a period of time. With the increasing assembly time, there is a possibility of slight desorption of molecules, followed by their rearrangement into a new stable state. Consequently, the SAM film formed may not exhibit the expected homogeneity and stability during prolonged self-assembly periods. This interpretation aligns with findings by Feng and colleagues. The CPE_f_ values also show a decreasing trend with increasing assembly time, indicating film thickening at the metal/solution interface. However, no correlation was observed between the assembly time and the CPE_dl_ variation. In all cases, the CPE values decreased with respect to that of the uncoated Cu, indicating an increase in the double layer thickness due to the formation of the SAM film at the metal/solution interface. Although the *n*_dl_ values remained nearly constant after film assembly, the *n*_f_ values were higher for the B-pollen/SAM-modified Cu electrodes. This outcome is attributed to the adsorption of B-pollen molecules on the Cu surface, leading to the formation of uniform, adherent, and low porosity B-pollen SAM films with respect to the uncoated Cu surface.

The Nyquist and Bode-phase plots of B-pollen/SAM electrodes assembled using different B-pollen concentrations (assembly time: 24 h, solvent: W) are shown in Figure 3c,c′, respectively. The corresponding fitting parameters of these curves are given in Table 2. The general shapes of the curves are similar to those obtained in the film assembly time and solvent optimization studies. As explained in detail earlier, the first capacitive loop corresponds to the CPE_dl_ – *R*_ct_.^80^ The second loop in the low-frequency region represents the film resistance and the accumulation of species at the metal/solution interface.

Clearly, the protective ability of the film is dependent on the B-pollen concentration in the film assembly medium. The total resistance, *R*_p_, increases with the B-pollen concentration up to 1000 ppm (Table 2). The highest resistance against corrosion and maximum η% were observed for the B-pollen/SAM-modified electrode fabricated at this concentration. At a higher B-pollen concentration (2000 ppm), the η% decreased again, which can be associated with a lower quality of the assembled film, as further discussed in subsequent characterization studies. In the concentration optimization, a scattering was observed in the log *f* – θ plots of the Cu electrodes modified at a 2000 ppm concentration and similarly in the time optimization after 48 h of modification. This scattering is due to the heterogeneous and nonuniform nature of the electrode surface. The reduced CPE values in the case of the film-modified electrodes are due to the presence of SAM films on the metal surface, which increases the double layer thickness. Overall, the data suggest that 1000 ppm of B-pollen is the optimal concentration for the preparation of B-pollen/SAM films to effectively inhibit the corrosion of Cu in NaCl solution.

#### 3.1.4 Linear Polarization Resistance

The electrochemical behavior of the SAM-modified electrodes, assembled under various conditions as previously described, was further evaluated using the LPR technique. Prior to measurement, the working was immersed in a 3.5% NaCl solution for 1 h to ensure a steady-state condition. Starting from more cathodic potentials ±0.01 V around *E*_ocp_, current–potential curves were obtained at a low scan rate. The *R*_p_ values of both uncoated Cu and the B-pollen/SAM-modified Cu electrodes were calculated from the slope of the polarization curves in the narrow potential range. The obtained values are given in Table 3.

**Table 3:** *R*_p_ and η% Values for Uncoated Cu and B-Pollen/SAM-Modified Cu Electrodes, Assembled under Varying Pollen Concentrations, Solvent Types, and Film Assembly Times, Calculated from the Linear Polarization Resistance Analysis

film assembly conditions			*R*_p_(Ω·cm^2^)	η%
uncoated Cu			188	-
solvent optimization	*C*: 1000 ppm, *t*: 24 h	E	1672	88.8
		**W**	**8337**	**97.7**
		M	5951	96.8
		AN	834	77.5
		A	2798	93.3
		EA	2399	92.9
		T	5364	96.5
		H	2196	91.4
concentration optimization	*t*: 24 h, solvent: W	100 ppm	5168	96.4
		250 ppm	5971	96.9
		500 ppm	6262	97.0
		**1000 ppm**	**8337**	**97.7**
		2000 ppm	3584	94.8
assembly time optimization	*C*: 1000 ppm, solvent: W	2 h	2635	92.9
		6 h	3954	95.2
		12 h	6392	97.1
		**24 h**	**8337**	**97.7**
		48 h	1461	87.7
		72 h	1193	84.2

The η% was calculated from the LPR data with the help of the equation (5, and the results are also given in Table 3

6 $$\eta \%=\frac{R_{p}^{\prime }-R_{p}}{R_{p}^{\prime }}\times 100$$

The *R*_p_ values obtained from the LPR results varied depending on the assembly time, solvent type, and B-pollen concentration, as shown in Table 3. According to the data, the η% values ranged from 97.7 to 77.5% depending on the solvent used. This variation can be attributed to the different physicochemical properties of the solvents. Complex and dynamic interactions among the metal surfaces, solvent molecules, and components of the B-pollen extract can significantly influence the film formation process.

It should also be noted that the composition of pollen extract is significantly dependent on the solvent used for extraction. This is another major factor affecting the protective performance of the assembled films. Additionally, the effect of film assembly time on the electrochemical behavior of the electrode is clearly demonstrated by the LPR analysis. The η% values calculated for different film formation durations ranged from 97.7 and 84.2%. Regarding the influence of B-pollen, the η% varied within the range of 97.7 to 94.8%.

The electrochemical data showed that the quality of the B-pollen/SAM films and their protective performance depends on the conditions of film assembly. The most suitable film for protecting Cu against corrosion in a 3.5% NaCl solution was assembled in water containing 1000 ppm of pollen after a 24 h assembly time.

### 3.2 Surface Characterizations of B-Pollen Films

The SEM image of the uncoated Cu electrode is shown in Figure 5a as the inset, revealing that the bare Cu surface has no film and exhibits scratches and abrasions from the sandpaper treatment. The AFM image of the unmodified Cu (inset in Figure 6a) confirms a comparable surface structure.^50^

**Figure 5:**
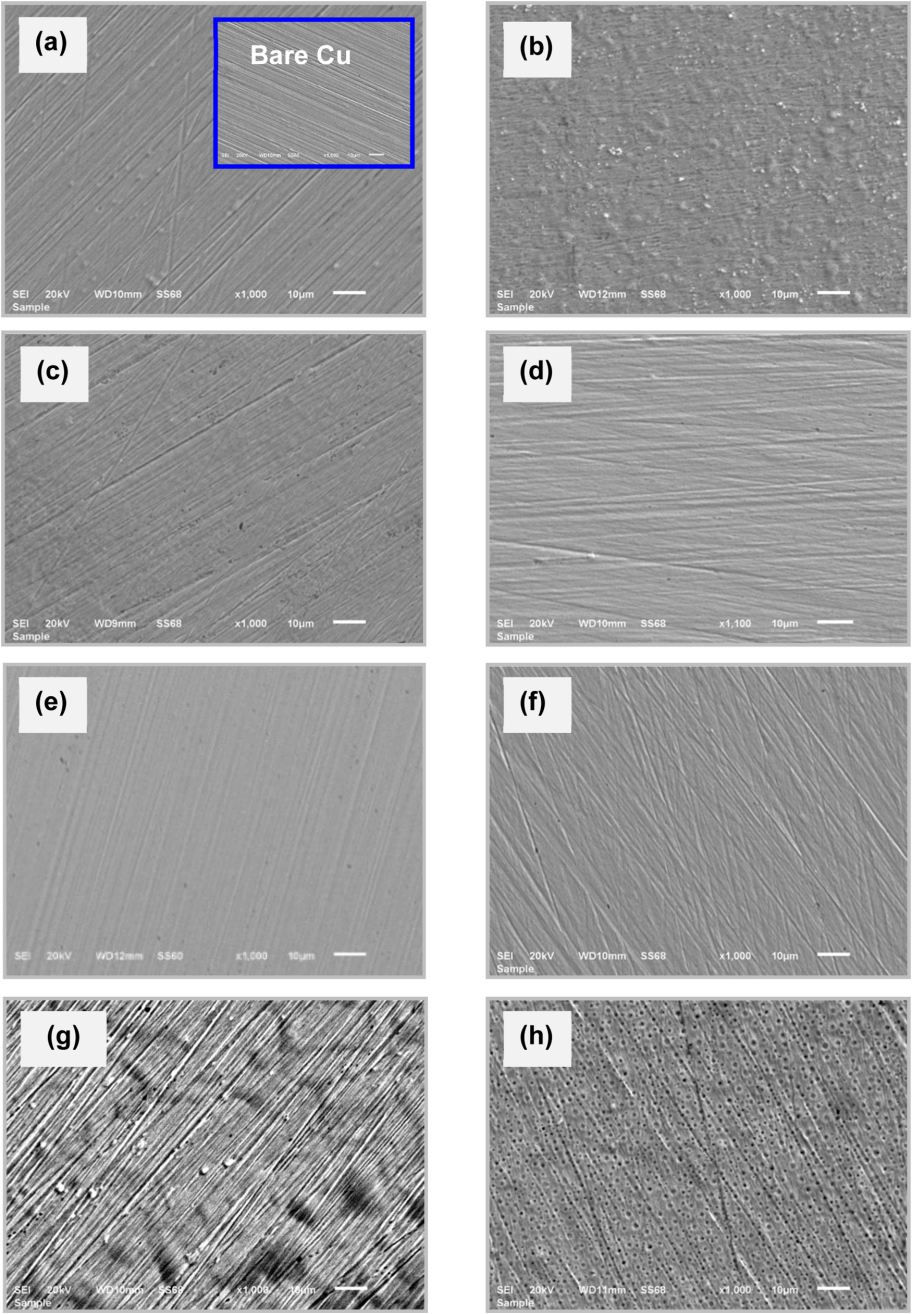
SEM images of the B-pollen/SAM-modified Cu electrodes prepared in different solvents with a 1000 ppm B-pollen concentration after a 24 h assembly time; E (a), AN (b), A (c), M (d), W (e), EA (f), H (g), and T (h). The inset in panel (a) shows the SEM image of the bare Cu surface (magnification: 1.000×; the length of the scale bar: 10 μm).

**Figure 6:**
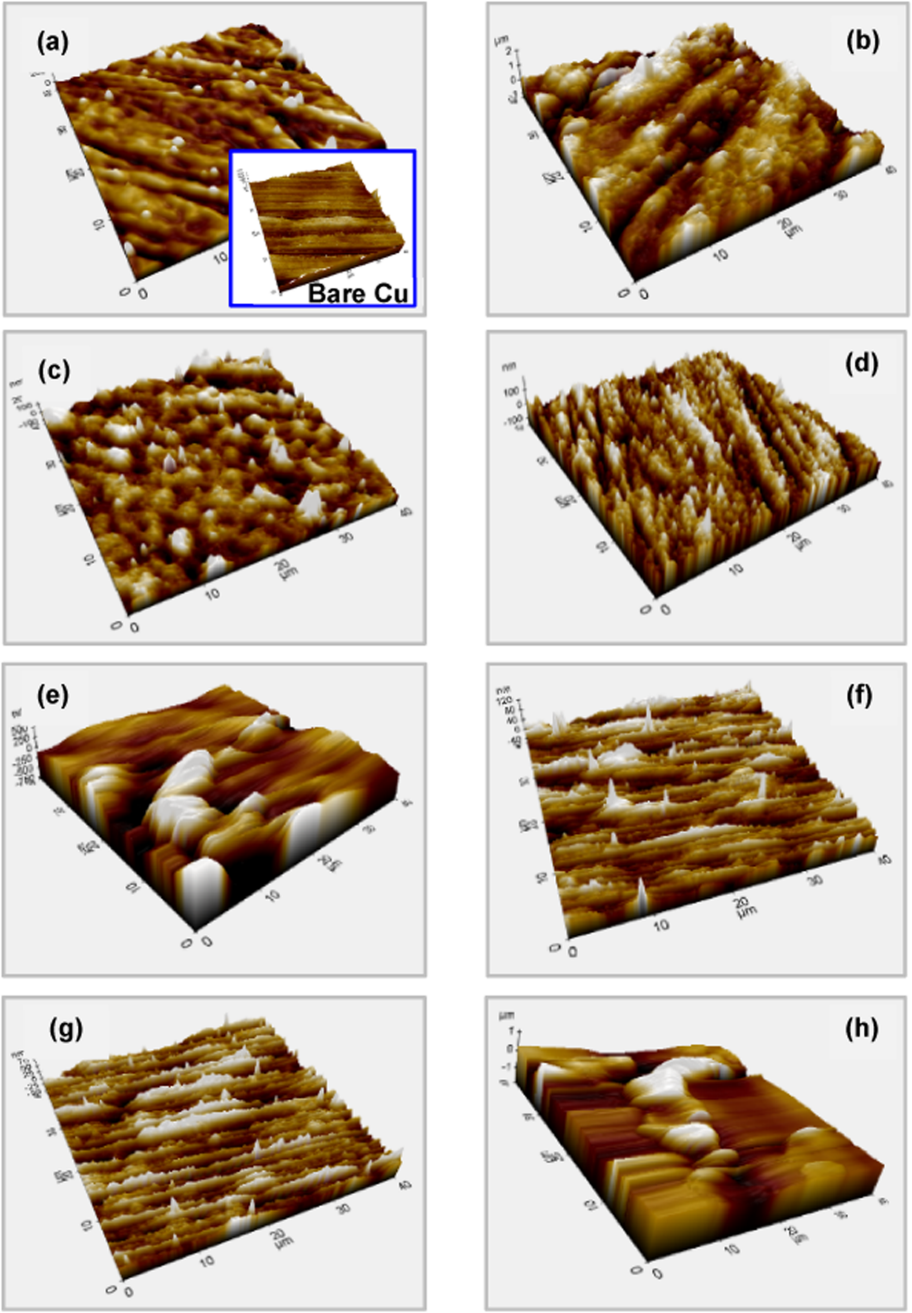
3D AFM images of the B-pollen/SAM-modified Cu electrodes prepared in different solvents with a 1000 ppm B-pollen concentration after a 24 h assembly time; E (a), AN (b), A (c), M (d), W (e), EA (f), H (g), and T (h). The inset in Figure 5a shows the 3d AFM image of the bare Cu surface.

Figure 5 shows SEM images of Cu electrodes assembled in different solvents with 1000 ppm of B-pollen concentration after a 24 h assembly time. The surface morphology of the modified Cu electrodes shows a relatively smooth surface, indicating that high-quality, compact, and tightly attached B-pollen/SAM films were successfully assembled on the Cu substrate under all optimized conditions. However, the quality and thickness of the films strongly depend on the assembly parameters. As depicted in Figure 5e, the films formed in W exhibit superior quality, making them more effective for corrosion inhibition applications.

The SAM images of B-pollen/SAM-modified Cu electrodes under various conditions, B-pollen concentrations (Figure S1), and film assembly times (Figure S1) are provided in Supporting Information. The surface properties of the resulting SAM films are significantly influenced by the duration of the film assembly (Figure S1).^81^ Figure S2 shows a notable improvement in the Cu surface after the film assembly with different B-pollen concentrations compared to that of the unmodified Cu electrode (Figure S1, inset). The disappearance of scratches and abrasions from the sandpaper indicates that the film has thickened. The thickest and highest-quality film was formed at a 1000 ppm B-pollen concentration, though further characterization studies are required to determine the exact thicknesses.

The SEM results were further supported by AFM studies (Figures 6, S3, and S4). As observed in these figures, the appearance and quality of the surface film depend on the assembly conditions. The best-quality SAM film, optimal for corrosion inhibition applications, was assembled in water using 1a 000 ppm B-pollen, with a 24 h assembly time. Since these details have been thoroughly discussed above, they will not be repeated here.

The hydrophobic/hydrophilic properties of the films were analyzed by using contact angle measurements, and the data obtained are shown in Table 4. Each measurement was repeated at least three times, at different surface zones, and the average values were used to generate the plots. As shown in Table 4, the uncoated Cu electrode had the lowest contact angle, indicating its hydrophilic nature. The increased contact angles observed after film modification showed that the B-pollen/SAM films enhance the hydrophobicity of the metal surface. As shown in Table 4, the hydrophobicity of the Cu surface increased after the film assembly in various solvents. According to Feng et al.,^82^ there is a correlation between the increased hydrophobicity and the delay in the arrival of corrosive ions at the metal surface, thereby enhancing corrosion resistance. It is important to note that the chemical composition of the B-pollen extract is strongly influenced by the solvent used, which, in turn, affects the surface properties of the film. Further chemical characterization studies are required to identify the precise molecular composition and individual effects of each component. However, this analysis falls outside the scope of this study and may be investigated in future work. The highest contact angle was observed for the film assembled over 24 h in the presence of 1000 ppm of B-pollen, confirming the optimal film assembly conditions for maximum corrosion protection.

**Table 4:** Contact Angles of Uncoated Copper and B-Pollen/SAM-Modified Copper Surfaces Prepared in Various Solvents, Concentrations, and Film Formation Times^a^

film assembly conditions	contact angle (°)							
uncoated Cu	55 ± 2.71							
solvent (*C*: 1000 ppm, *t*: 24 h)	E	M	W	A	AN	EAs	T	H
	70 ± 0.270	78 ± 0.87	80 ± 0.31	81 ± 0.72	87 ± 0.92	93 ± 1.74	95 ± 0.67	85 ± 1.42
film formation time (solvent: W, *C*: 1000 ppm)	2 h	6 h	12 h	24 h	48 h	72 h		
	73 ± 0.95	74 ± 0.48	66 ± 1.08	80 ± 0.31	78 ± 0.90	80 ± 0.63		
concentration (solvent: W, *t*: 24 h)	100 ppm	250 ppm	500 ppm	1000 ppm	2000 ppm			
	57 ± 1.7	76 ± 0.24	46 ± 3.74	80 ± 0.31	63 ± 0.36			

a C: concentration of B-pollen, t: film formation time, W: water.

### 3.3 Analysis of Phenolic Compounds in Bingöl Pollen

The chemical composition of B-pollen extracted in water is previously shown in Table 5. A quantitative analysis of its hydrophilic component revealed 36 potential anticorrosive compounds. Among these, eriodictyol (3604.1 μg PC/g DE) was identified as the primary component, with notable quantities of narcissin (91.8 μg PC/g DE) and afzelin (51.8 μg PC/g DE). Flavonoid compounds such as eriodictyol, narcissin, and afzelin, detected in Bingöl pollen, belong to a group of secondary metabolites commonly found in many natural sources such as different bee pollen, fruits, and vegetables. These compounds are known to provide broad-spectrum health benefits.^83^ Additionally, they are classified as environmentally friendly corrosion inhibitors, similar to other natural product-derived corrosion inhibitors.^84^ Since flavonoids are predominantly derived from natural sources, they are considered suitable for use as corrosion inhibitors, provided their molecular structures support such a functionality.^19^

**Table 5:** Phenolic Compounds of Bingöl Pollen^a^

phenolic compounds	RT*	chemical formula	molecular ion (*m*/*z*)	amount (μg PC/g DE)	*R* ^2^	LOD	LOQ
arbutin	0.64	C_12_H_16_O_7_	271.08233	3.4	0.9928	0.80	2.67
quinic acid	1.17	C_7_H_12_O_6_	191.05611	6.2	0.9912	0.28	0.94
gallic acid	2.17	C_7_H_6_O_5_	169.01425	1.6	0.9963	0.04	0.13
protocatechuic acid	4.38	C_7_H_6_O_4_	153.01933	0.7	0.9990	0.26	0.85
3,4-dihydroxyphenylacetic acid	5.38	C_8_H_8_O_4_	167.03498	22.5	0.9934	0.32	1.08
esculin hydrate	6.17	C_15_H_16_O_9_ xH_2_O	339.07216	0.7	0.9960	0.26	0.85
4-hydroxybenzoic acid	6.25	C_7_H_6_O_3_	137.02442	0.8	0.9953	0.29	0.97
chlorogenic acid	6.86	C_16_H_18_O_9_	353.08781	3.3	0.9949	1.17	3.91
benzoic acid	7.00	C_7_H_6_O_2_	121.02950	27.8	0.9933	0.68	2.27
caffeic acid	7.24	C_8_H_9_O_4_	179.03498	7.3	0.9973	0.17	0.57
quercetin 3-rutinoside 7-glucoside	7.25	C_33_H_40_O_21_	771.20374	0.7	0.9937	1.63	5.43
syringic acid	7.41	C_9_H_10_O_5_	197.04555	0.5	0.9903	0.55	1.82
3-(4-hydroxyphenyl) propionic acid	7.69	C_9_H_10_O_3_	165.05572	11.4	0.9989	3.40	11.33
vicenin 2	7.82	C_27_H_30_O_15_	593.15119	16.6	0.9947	0.75	2.51
ethylgallate	7.96	C_9_H_10_O_5_	197.04555	0.1	0.9919	0.23	0.78
coumaric acid	8.32	C_9_H_8_O_3_	163.04007	2.3	0.9949	0.26	0.87
schaftoside	8.34	C_26_H_28_O_14_	563.14063	0.4	0.9935	1.13	3.76
luteoloside	8.46	C_21_H_20_O_11_	449.10784	21.9	0.9930	4.40	14.67
eriodictyol	8.46	C_15_H_12_O_6_	287.05501	3604.1	0.9927	0.86	2.87
luteolin-7-rutinoside	9.09	C_27_H_30_O_15_	595.16364	11.9	0.9966	0.48	1.58
rutin hydrate	9.19	C_27_H_30_O_16_ xH_2_O	609.14611	36.9	0.9931	0.49	1.65
luteolin-7-*O*-glucuronide	9.26	C_27_H_30_O_15_	461.07255	0.3	0.9916	0.21	0.69
isoquercitrin	9.26	C_21_H_20_O_12_	463.08820	12.6	0.9924	0.45	1.51
hyperoside	9.26	C_21_H_20_O_12_	463.08820	12.8	0.9904	0.24	0.79
myricetin	9.40	C_15_H_10_O_8_	319.04291	10.9	0.9936	0.47	1.58
ellagic acid	9.43	C_14_H_6_O_8_	300.99899	0.8	0.9939	1.39	4.65
rosmarinic acid	9.55	C_18_H_16_O_8_	359.07724	0.4	0.9968	0.56	1.88
nicotiflorin	9.72	C_27_H_30_O_15_	593.15106	3.0	0.9815	2.67	8.90
astragalin	9.74	C_21_H_20_O_11_	447.09328	9.0	0.9903	0.84	2.80
kuromanine	9.74	C_21_H_21_ClO_11_	447.09328	9.5	0.9917	0.47	1.57
narcissin	9.81	C_28_H_32_O_16_	623.16176	91.8	0.9918	1.00	3.35
afzelin	10.30	C_21_H_20_O_10_	431.09837	51.8	0.9983	0.42	1.39
kaempferol	10.30	C_15_H_10_O_6_	287.05350	28.3	0.9945	0.72	2.40
quercetin	10.50	C_15_H_10_O_7_	301.03538	9.3	0.9958	3.50	11.67
luteolin	10.80	C_15_H_10_O_6_	285.04046	7.8	0.9963	1.33	4.43
isorhamnetin	11.30	C_16_H_12_O_7_	315.05103	5.4	0.9936	1.34	4.46

a Dried extract (DE), phenolic compound (PC), retention time (RT), limit of detection (LOD), limit of quantitation (LOQ), and correlation coefficient (*R* ^2^).

As shown in Table 5, B-pollen contains a diverse range of phenolic components, leading to film formation through a mixture of these components. It is important to note that the chemical composition of B-pollen is highly dependent on the solvent applied for extraction. However, since solvent effects were not the primary focus of this study and the research was already extensive, only the ethanol extract was analyzed as a representative sample.

The interactions between these phenolic components and their interactions with solvent molecules significantly impact the quality of the self-assembled films. Determining which specific components contribute most to film formation would require an individualized analysis of each compound. However, such a detailed investigation falls beyond the scope of this study. Instead, the study focused on the practical evaluation of the film properties formed by the B-pollen extract and its inhibitory effect on the corrosion of copper in a 3.5% NaCl solution.

## 4 Conclusions

In this research, thin, adherent, compact, and uniform B-pollen/SAM films were successfully self-assembled on Cu surface under various conditions, including solvent type, B-pollen concentration, and film assembly durations. The optimal conditions for the preparation of these films were identified to achieve effective corrosion inhibition of Cu in a 3.5% NaCl solution. The films were characterized using various surface analysis techniques, including SEM, AFM, and contact angle measurements. The key findings of the study are summarized below:

- Surface analysis revealed the presence of dense, thin, compact, and adhesive B-pollen/SAM films on the Cu surface.
- B-pollen/SAM films, prepared under different optimized conditions, effectively mitigated the corrosion rate of Cu in a 3.5% NaCl solution.
- B-pollen/SAM films provided corrosion protection primarily through a physical barrier effect, without altering the mechanism of Cu dissolution.
- The film quality and protective performance are significantly influenced by the assembly conditions.
- The most effective corrosion protection was achieved using water as the solvent, with 1000 ppm of B-pollen after 24 h of film assembly. Under these optimized conditions, the corrosion inhibition efficiency of the B-pollen/SAM reached 98.45%.

These results highlight the potential of B-pollen/SAM films as an environmentally friendly and effective corrosion inhibition strategy, offering a sustainable approach to Cu protection. This study provides a strong experimental foundation for the future design and development of new self-assembling molecules from natural organic products. However, a notable challenge in this study was the complexity of the chemical composition of pollen, which contains a large number of interacting components. Understanding how these components interact with each other and with the metal surface requires further investigation and will be the focus of a future study. In addition, surface imaging showed that the films were not uniformly monolayered in some places, which requires further analysis, although this was not the primary focus of this study.

## Supplementary Material



## Notes

SEM images of B-pollen/SAM-modified Cu electrodes prepared at different assembly times (Figure S1); SEM images of B-pollen/SAM-modified Cu electrodes prepared at different B-pollen concentrations (Figure S2); 3D AFM images of B-pollen/SAM-modified Cu electrodes prepared at different assembly times (Figure S3); and 3D AFM images of B-pollen/SAM-modified Cu electrodes prepared at different B-pollen concentrations (Figure S4) (PDF)
