# Supplementary material for: Bingöl Pollen Self-Assembled Natural Thin Films: Fabrication, Characterization, and Corrosion Inhibition Performance for Copper Protection in a NaCl Environment
Source: ACS Omega. 2025 May 27;10(22):23281–96. doi: 10.1021/acsomega.5c01646 (PMC12163765; doi:10.1021/acsomega.5c01646)
Supplement: Supplementary file 1 [file ao5c01646_si_001.pdf]

Bingöl pollen self-assembled natural thin films: Fabrication, characterization and corrosion inhibition performance for copper protection in a NaCl environment

Ramazan Solmaz*, [a], Ece Altunbaş Şahin*, [b], Yeşim Aydın Dursun[a,c], Yakubu Sawadogo Adam[d], İbrahim Halil Gecibesler[a], Mustafa Doğrubaş[e], Nevzat Çağlayan[f], İbrahim Y. Erdoğan[a], Sinan Bayındır[g], Gülfeza Kardaş[d]

[a]*Bingöl University, Health Sciences Faculty, Occupational Health and Safety Department, 12000, Bingöl, Türkiye*
[b]*Bingöl University, Genç Vocational School, Property Protection and Security Department, Civil Defense and Firefighting Program, 12000, Bingöl, Türkiye*
[c]*Bingöl University, Graduate School of Natural and Applied Sciences, Chemistry Department, Bingöl, Türkiye*
[d]*Çukurova University, Science and Letters Faculty, Chemistry Department, 01330, Adana, Türkiye*
[e]*Bingöl University, Graduate School of Natural and Applied Sciences, Occupational Health and Safety Department, Bingöl, Türkiye*
[f]*Bingöl University, Vocational School of Food, Agriculture, and Livestock, Plant and Animal Production Department, Beekeeping Program, 12000, Bingöl, Türkiye*
[g]*Bingöl University, Science and Letters Faculty, Chemistry Department, 12000, Bingöl, Türkiye*

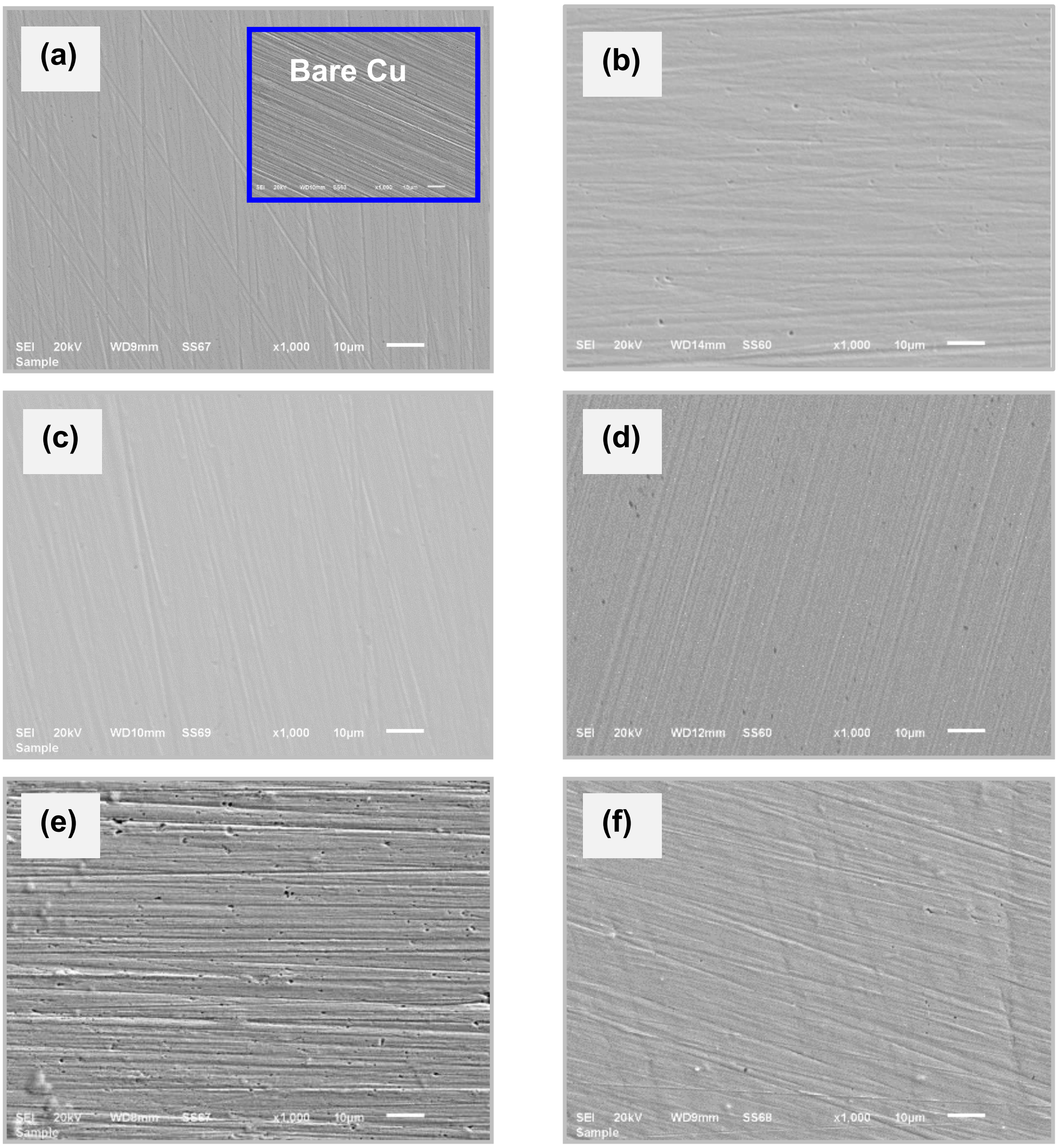

Figure S1. SEM images of the B-pollen/SAM modified Cu electrodes prepared in water with 1000 ppm B-pollen concentration after different film assembly times; 2 h (a), 6 h (b), 12 h (c), 24 h (d) 48 h (e), 72 h (f). The inset in Figure S1 (a) shows SEM image of bare Cu surface (Magnification: 1.000x, The length of scale bar: 10 µm)

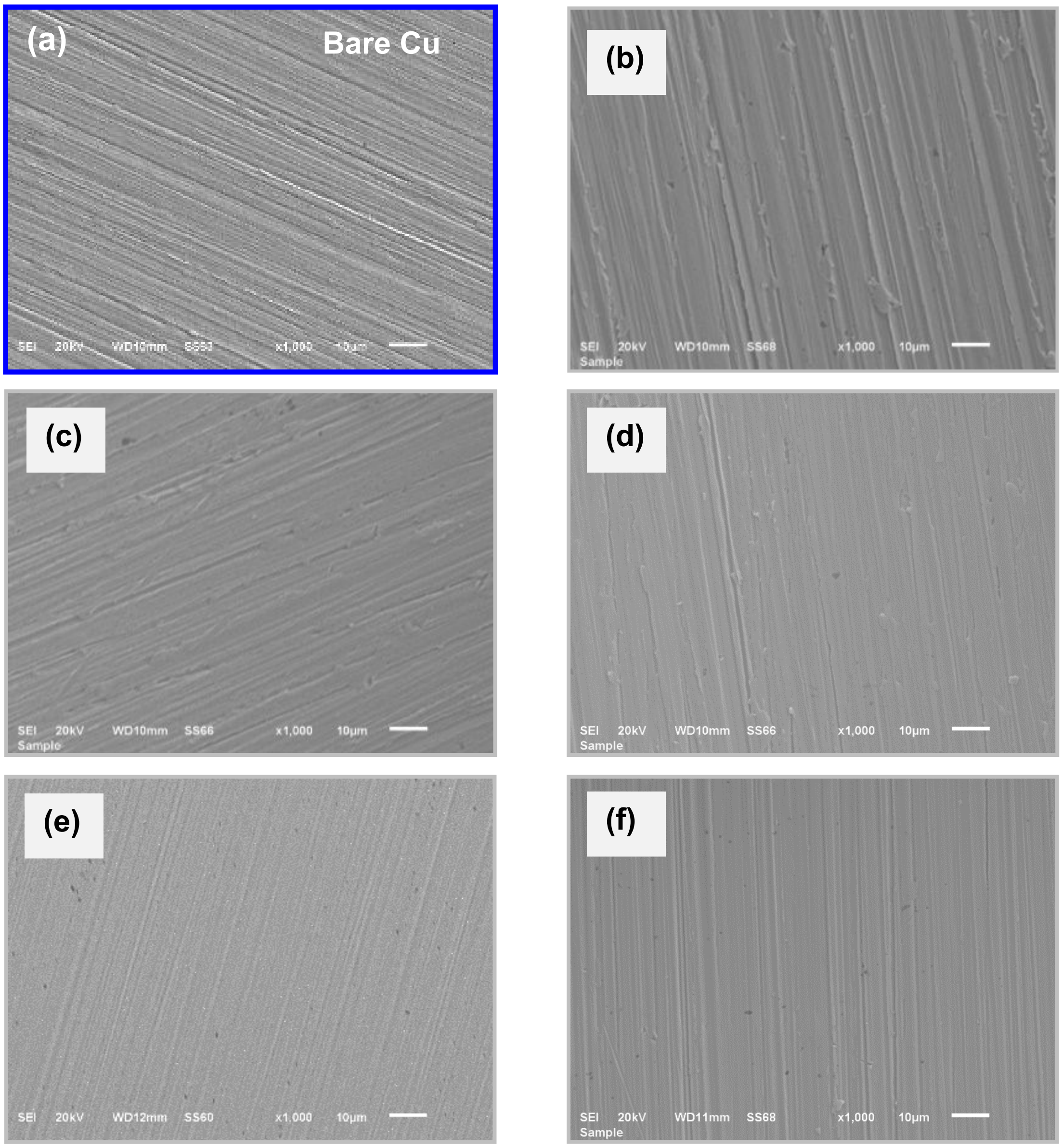

Figure S2. SEM images of the B-pollen/SAM modified Cu electrodes prepared in water with different B-pollen concentrations after 24 hours assembly time; bare Cu (a), 100 ppm (b), 250 ppm (c), 500 ppm (d) 1000 ppm (e), 2000 ppm (f). (Magnification: 1.000x, The length of scale bar: 10 µm)

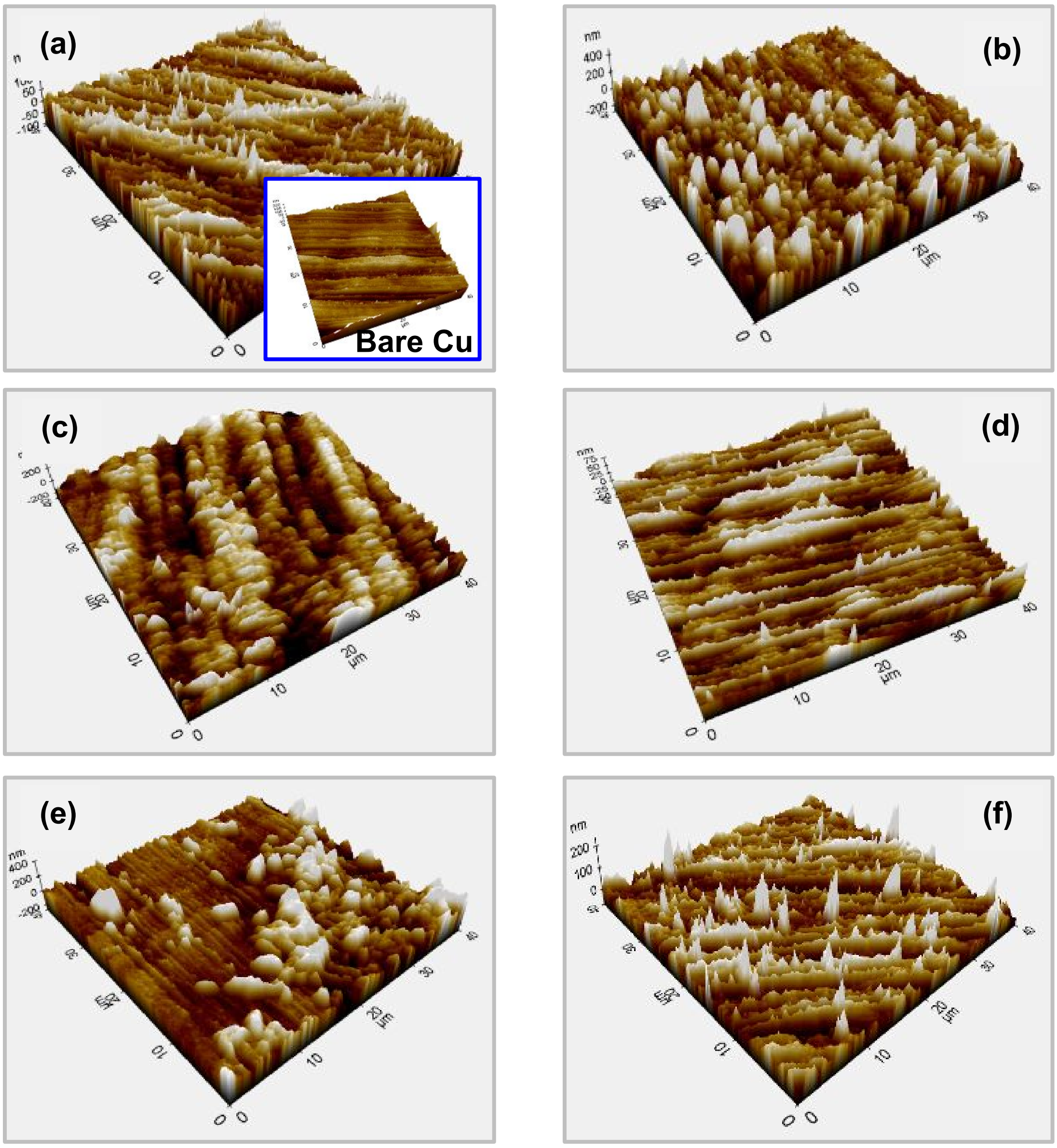

Figure S3. 3D AFM images of the B-pollen/SAM modified Cu electrodes prepared in water with 1000 ppm B-pollen concentration after different film assembly times; 2 h (a), 6 h (b), 12 h (c), 24 h (d) 48 h (e), 72 h (f). The inset in Figure S1 (a) shows AFM image of bare Cu surface

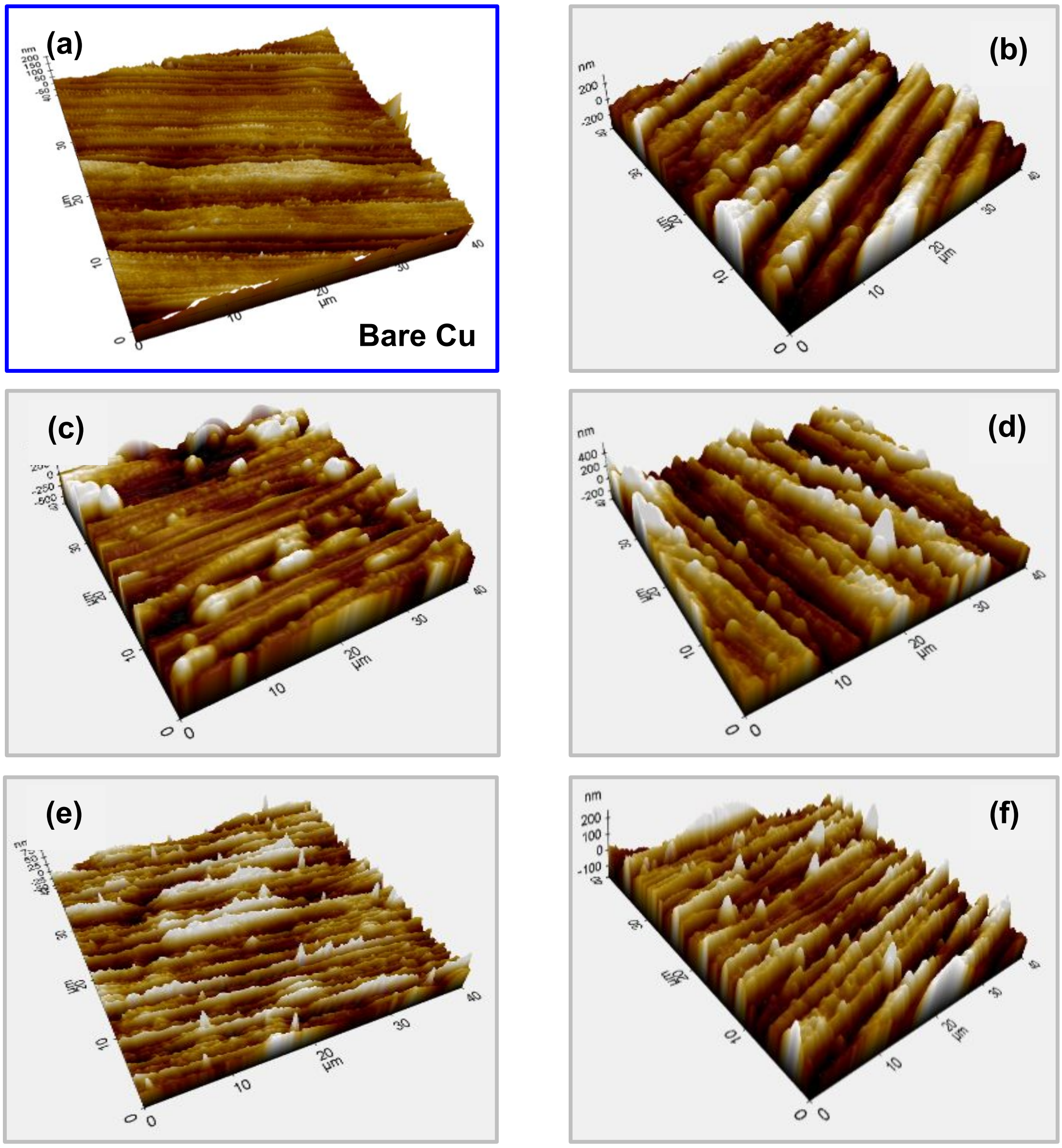

Figure S4. 3D AFM images of the B-pollen/SAM modified Cu electrodes prepared in water with different B-pollen concentrations after 24 hours assembly time; bare Cu (a), 100 ppm (b), 250 ppm (c), 500 ppm (d) 1000 ppm (e), 2000 ppm (f)
